# Exploring Associations Between STEAM-Based Interventions and Executive and Cognitive Skills in Children with ADHD

**DOI:** 10.3390/healthcare14020169

**Published:** 2026-01-08

**Authors:** María del Mar Bueno-Galán, Carlos Barbosa-Torres, María José Godoy-Merino, Alperen Yandi, Alejandro Arévalo-Martínez, María Pilar Cantillo-Cordero, María Elena García-Baamonde Sánchez, Juan Manuel Moreno-Manso

**Affiliations:** 1 Department of Educational Science, Universidad de Extremadura, 06006 Badajoz, Spain; marbueno@unex.es (M.d.M.B.-G.); godoymerino21@unex.es (M.J.G.-M.); 2 Department of Psychology, Universidad de Extremadura, 06006 Badajoz, Spain; aarevaloj@unex.es (A.A.-M.); pcantillo@unex.es (M.P.C.-C.); mgarsan@unex.es (M.E.G.-B.S.); jmmanso@unex.es (J.M.M.-M.); 3 Department of Education, Bolu Abant Izzet Baysal University, Bolu 14030, Turkey; alperenyandi@gmail.com

**Keywords:** executive functions, cognitive skills, ADHD, quality of life, daily life, STEAM

## Abstract

**Highlights:**

**What are the main findings?**

- The executive functions and cognitive skills of children diagnosed with ADHD who participated in STEAM-based interventions were significantly improved.
- Gains were observed in inhibition, planning, flexibility, verbal comprehension, visuospatial abilities, processing speed, and impulse control.

**What are the implications of the main findings?**

- The lives of children with ADHD may be positively impacted by the benefits that STEAM-based methodologies offer in strengthening their executive functions and cognitive skills.
- These findings contribute to the development of inclusive pedagogical strategies aimed at improving the daily functioning and overall well-being of neurodivergent populations.

**Abstract:**

**Background**: This study examines whether participation in STEAM-based educational activities is associated with improvements in executive functions (EFs) and cognitive skills in children with Attention Deficit Hyperactivity Disorder (ADHD). **Methods**: A total of 60 children diagnosed with ADHD (mean age = 8 years) participated, with 30 following a traditional educational approach and 30 engaged in STEAM-based activities. Executive functions and cognitive abilities were assessed using standardized instruments (BRIEF, WISC-V, CARAS-R), and data were analyzed with IBM SPSS Statistics 25. **Results**: Children in the STEAM group outperformed the control group across several domains, showing statistically significant gains in inhibition, planning and organization, verbal comprehension, visuospatial skills, processing speed, total IQ, efficiency, and the Impulsivity Control Index (ICI). **Conclusions**: These findings suggest that STEAM-based educational experiences may support neurodevelopmental growth and enhance cognitive and executive functioning in children with ADHD, although causal inferences cannot be drawn due to the cross-sectional design.

## 1. Introduction

Attention Deficit Hyperactivity Disorder (ADHD) is a psychopathological condition characterized by a persistent pattern of inattention, hyperactivity, and impulsivity [1,2]. It is frequently accompanied by psychiatric comorbidities such as depression, bipolar disorder, and anxiety, as well as medical conditions including epilepsy, sleep disorders, concussions, chromosomal abnormalities, and motor immaturity [3]. The impact of ADHD extends across cognitive, academic, social, and emotional domains [4], significantly reducing quality of life [3]. Deficits in cognitive abilities and executive functions [1], together with language-related difficulties, contribute to poor academic performance [5], disruptive behaviors, and challenges in interpersonal relationships. Difficulties in sustaining attention, switching between tasks, resisting distractions, and adapting to changing demands are also common [2,4,5,6].

Executive functions (EFs) and related cognitive skills are fundamental to adaptive functioning in daily life. Adequate EF performance supports the autonomous and effective execution of daily tasks [7], while strong working memory contributes to better emotional regulation [8]. Improvements in inhibition can enhance this regulation [9], and greater development in planning, attention, and memory is associated with more frequent use of adaptive emotional regulation strategies [10]. In this context, verbal comprehension, processing speed, visuospatial skills, and impulse control constitute examples of cognitive abilities that influence the daily functioning of children with ADHD. Strong verbal comprehension facilitates the retention and integration of orally presented information, enabling children to follow multi-step instructions, participate in classroom discussions, and engage in adaptive social interactions [11]. Processing speed influences the ability to complete academic tasks within time constraints, respond promptly in social exchanges, and keep pace with classroom demands; slower processing speed is associated with reduced responsiveness to interventions and greater frustration [12]. Visuospatial skills underpin practical competencies such as organizing materials, navigating environments, and interpreting visual information like maps or diagrams [13]. Finally, impulse control supports appropriate behavior in social contexts, fosters positive peer relationships, and promotes autonomy by enabling children to follow routines, complete tasks independently, and make considered decisions in daily life [14]. Fostering these abilities not only strengthens emotional regulation but also enhances emotional competence [15,16]. Cognitive skills are positively associated with students’ academic development [17], and more developed EFs are linked to better academic outcomes [18,19,20,21].

In recent years, STEAM education, integrating Science, Technology, Engineering, Arts, and Mathematics, has emerged as an effective approach for developing competencies such as sustained attention, working memory, planning, and cognitive flexibility, all of which are essential for meeting the demands of daily life [22,23,24,25,26,27].

Participation in STEAM programs has been associated with improvements in self-regulation, problem-solving, and collaborative work, which benefit all children by fostering autonomy, social adaptation, and quality of life. These areas are particularly significant for children with ADHD, as they support the regulation of executive functions, enhance motivation, and promote more adaptive social and emotional behaviors [28,29,30,31].

In most studies examining the effects of STEAM-based activities on executive functions, participants are typically developing children, and significant improvements have been reported following the implementation of such methodologies. Notable differences in working memory have been observed following STEAM interventions [31,32,33,34,35]. In the domain of visuospatial skills, significant gains were identified in studies by [30,32]. Inhibition has also shown measurable improvements, particularly in the findings of [31,36]. Planning abilities were positively impacted in studies such as [37], while cognitive flexibility was enhanced according to [38]. Although these findings support the relevance of STEAM-based interventions in strengthening specific components of executive functioning, it is important to note that they have been primarily observed in children with typical developmental trajectories.

Other authors have reported positive effects of educational interventions in students with learning difficulties [39], this study only emphasizes the significant potential of STEAM activities to enhance learning, engagement, and social interaction in children with neurodevelopmental disorders; it does not focus on specific executive functions. The same applies to [35], who examined the effects of STEAM activities in children with learning difficulties, but not exclusively in a sample composed of children with ADHD. Few studies have specifically examined the benefits of STEAM in ADHD populations. Other authors [40] report that STEAM-based interventions are beneficial for executive functioning in children with ADHD, although they do not specify which components are affected. Building on this, another study [41] documented significant improvements in sustained attention, inhibition, planning, and impulse control following the implementation of STEAM-based interventions, specifically those involving educational robotics.

Cognitive training interventions, such as STEAM programs [42], can enhance executive functions during childhood. Strengthening cognitive skills is particularly relevant for children with ADHD, as these skills underpin more efficient executive functioning [43,44,45] and can promote a better quality of life [40]. Despite growing evidence of the cognitive and executive benefits of STEAM-based interventions in typically developing children, research on their effectiveness for children with ADHD remains scarce. Given that ADHD is characterized by deficits in attention, working memory, inhibition, and other EFs, STEAM activities represent a promising avenue for enhancing these skills through engaging, hands-on, and cognitively stimulating experiences, with potential benefits for social interaction and self-esteem.

Consequently, the present study aims to analyze the impact on the development of executive functions and cognitive skills in a sample of children with ADHD, resulting from the implementation of an educational approach grounded in STEAM-based experiences.

## 2. Materials and Methods

### 2.1. Research Model and Procedure

This study employed a quasi-experimental, cross-sectional comparative design to examine differences in executive functions and cognitive abilities between students following a traditional methodology and those engaged in an educational program grounded in STEAM principles. The STEAM-based program, implemented at a Psychopedagogical and Educational Innovation Center in Badajoz, Spain, included structured activities aimed at engaging core executive processes such as planning, working memory, cognitive flexibility, and inhibitory control. The program was delivered four days per week, with one-hour sessions each day, and its main characteristics were: (A) on the first day children conducted experiments to explore science content; (B) on the second day they engaged in robotics and coding using LEGO kits, Scratch, or Minecraft to integrate technology and engineering; (C) on the third day they worked on mathematics through real-world problem solving; (D) on the fourth day they focused on art by designing and decorating creations and models produced in the robotics or engineering workshops. All activities delivered primary school curricular content through robotics, coding, programming, and experiential learning, connecting academic concepts to students’ everyday context in an inquiry-based manner. In contrast, children in the traditional methodology group covered the same curricular content of the different subjects through textbooks and teacher-led explanations, following a conventional instructional approach. Participants in the STEAM group had been enrolled at the center prior to the study, resulting in varied exposure durations (M = 3.93 years, SD = 1.28). The procedure comprised three phases: recruitment and group assignment, implementation of the program, and assessment of executive functions and cognitive abilities. Data collection occurred at a single time point for both groups. Executive functions were assessed using standardized instruments (BRIEF, WISC-V, CARAS-R) specifically designed to measure these processes, and all assessments were administered individually under controlled conditions by trained evaluators who were educational psychologists specialized in psychopedagogical assessment and had more than 10 years of professional experience.

### 2.2. Research Context and Sample

The sample consisted of 60 children divided into two groups: 30 children following a traditional educational methodology (control group) and 30 children who had participated in the STEAM program. Participants were recruited from the center’s client base and met the inclusion criteria of being within the target age range and having a diagnosis of ADHD. Group assignment was not randomized; children were included in either the STEAM or control group based on their prior participation in the center’s programs. Descriptive statistics of the participants, including gender, age, and years of exposure to the STEAM program, are presented in Table 1.

### 2.3. Instruments

- Behavior Rating Inventory of Executive Function (BRIEF):

To assess executive functioning in children and adolescents, the Behavior Rating Inventory of Executive Function (BRIEF), adapted into Spanish by TEA Editions [46], was employed. In the present study, the parent version was used. The questionnaire provides scores across nine clinical scales, which include inhibition, self-monitoring, flexibility, emotional control, initiative, working memory, planning and organization, task supervision, and organization of materials. On the BRIEF scale, lower scores indicate better executive functioning.

The Spanish adaptation of the BRIEF-2 demonstrates high internal consistency and excellent reliability, along with a validated structure and strong correlations with other measures, supporting its utility in both clinical and educational settings [47].

The BRIEF provides a detailed picture of executive functioning. Success in inhibition is directly related to better impulse control and more appropriate behavior in social and academic contexts. Self-monitoring evaluates the child’s awareness of the impact of their own behavior on others. Flexibility assesses cognitive flexibility, and achievement in this domain implies effective shifting between tasks and stronger problem-solving skills. Emotional control reflects the regulation of emotional responses. Finally, initiative, working memory, planning and organization, together with task supervision, are closely linked to attentional capacity, problem-solving, and the ability to order, prioritize, and set goals [46].

- Wechsler Intelligence Scale for Children―Fifth Edition (WISC-V):

Cognitive abilities were assessed using the Wechsler Intelligence Scale for Children Fifth Edition [48]. The WISC-V provides a Full-Scale IQ (FSIQ), which represents general intellectual ability (g), and five primary index scores: Verbal Comprehension, Visual–Spatial, Fluid Reasoning, Working Memory, and Processing Speed [49,50].

Performance on the WISC-V indices provides important insights into cognitive functioning. Success in verbal comprehension tasks is closely related to cognitive flexibility, long-term memory, lexical knowledge, and verbal expression, all of which support adaptive communication and learning. High achievement in visuospatial tasks reflects general visual intelligence, perceptual accuracy, organizational skills, and visuomotor coordination, competencies that are essential for academic and everyday functioning. Similarly, strong results in fluid reasoning are associated with visual intelligence, spatial capacity, classification skills, and also with working memory and attentional control, highlighting the integrative nature of reasoning processes. Success in working memory tasks is linked to cognitive flexibility, mental agility, and memory consolidation, while faster processing speed is connected to attentional focus, concentration, motivation, rapid decision-making, and inhibitory control. Taken together, these associations indicate that improvements across WISC-V domains are not isolated but converge to enhance overall learning capacity [48].

- Test CARAS-R:

The CARAS-R test [51] is a brief and reliable psychometric tool used to assess selective attention and impulsivity control in children and adolescents. It takes approximately three minutes to administer and can be applied individually or in groups. Its technical quality is supported by Hogrefe TEA Editions, which provides updated norms and scoring procedures for various educational and clinical contexts. High scores in Accuracy and in the Impulsivity Control Index (ICI) indicate stronger sustained attention and better inhibitory control.

Its validity has been confirmed in multiple studies. Some authors [52] demonstrated its effectiveness in measuring sustained attention and identifying impulsive response patterns in Spanish children. Several researchers [53] validated the attentional efficacy index (EA) in Argentine students, while another [54] showed a perfect correlation between EA and the impulsivity control index (ICI), allowing for cross-population score conversion.

### 2.4. Data Analysis

Data were analyzed using IBM SPSS Statistics 25 [55]. Normality was assessed using the Shapiro–Wilk test, and homogeneity of variances was evaluated with Levene’s test. Since all variables met the assumptions of normality and homogeneity, independent samples *t*-tests were applied to compare the control and STEAM groups. Effect sizes (Cohen’s d) were also calculated to determine the magnitude and precision of the observed differences.

## 3. Results

Descriptive statistics for the BRIEF indices are presented in Table 2. In these scales, lower scores indicate better executive functioning, whereas higher scores reflect greater difficulties. Descriptive analyses revealed that the STEAM group showed lower mean scores compared to the control group in several subscales, including Inhibition (STEAM: 54.60 vs. Control: 61.13), Emotional Control (STEAM: 53.93 vs. Control: 54.40), Working Memory (STEAM: 59.80 vs. Control: 62.53), Planning and Organization (STEAM: 56.00 vs. Control: 61.27), Task Supervision (STEAM: 57.33 vs. Control: 59.47), Behavioral Regulation Index (STEAM: 57.47 vs. Control: 60.20), Cognitive Regulation Index (STEAM: 58.80 vs. Control: 61.27), and the Global Executive Index (STEAM: 59.93 vs. Control: 61.47).

Appropriate tests were conducted to assess the assumption of normality, and results indicated that this assumption was not met. Consequently, the non-parametric Mann–Whitney U test was applied across all scales. Statistically significant differences were found in the Inhibition (*p* = 0.028), Flexibility (*p* = 0.050), and Planning and organization (*p* = 0.047). Other scales, such as self-monitoring, did not reach statistical significance, although they approached the threshold closely (*p* = 0.057). Cohen’s d was also calculated to determine the effect size and revealed effects ranging from moderate (such as in the cases of flexibility, self-monitoring, and planning and organization) to large, as observed in inhibition.

Descriptive statistics for the WISC-V indices are presented in Table 3. In these scales, higher scores represent better cognitive performance, whereas lower scores indicate weaker abilities in the respective domains. Across all indices, the STEAM group obtained higher mean scores compared to the control group. Specifically, the Verbal Comprehension Index averaged 112.13 in the STEAM group versus 101.47 in the control group. The Visuospatial Index showed the largest difference, with means of 114.33 and 97.07, respectively. Similarly, the Fluent Reasoning Index, Working Memory Index, and Processing Speed Index were higher in the STEAM group (107.40 vs. 101.80; 101.00 vs. 98.13; 92.67 vs. 81.80, respectively). Overall, the Total IQ score was also elevated in the STEAM group (106.73) relative to the control group (95.27).

Some indices met the assumption of normality, while others did not. Accordingly, either parametric or non-parametric tests were applied, depending on the characteristics of each case. Statistically significant differences were found in the Verbal Comprehension Index (*p* = 0.012), Visuospatial Index (*p* = 0.000), Processing Speed Index (*p* = 0.006), and Total IQ (*p* = 0.000).

Cohen’s d values were calculated for each WISC-V index to estimate the effect sizes associated with participation in the STEAM program compared to the control group. The results revealed large effect sizes for Verbal Comprehension (d = 0.79), Processing Speed (d = 0.79), and Total IQ (d = 1.05), as well as a very large effect for the Visuospatial Index (d = 1.28). Moderate and small effects were observed for Fluent Reasoning (d = 0.42) and Working Memory (d = 0.17), respectively. These results can be seen in Table 4.

Descriptive statistics for the CARAS-R measures are presented in Table 5. In this test, higher scores in Accuracy and Impulsivity Control Index (ICI) indicate better performance, whereas lower scores reflect poorer outcomes. For the Accuracy measure, the control group (*n* = 30) obtained a mean of 3.13 (SD = 1.57), whereas the STEAM group (*n* = 30) showed a higher mean of 5.20 (SD = 1.71). Regarding the Impulsivity Control Index (ICI), the control group scored a mean of 2.40 (SD = 1.52), while the STEAM group obtained a higher mean of 4.53 (SD = 1.38).

Accuracy and the impulsivity control index did not meet the normality assumption; consequently, a non-parametric Mann–Whitney U test was conducted for this measure. Statistically significant differences between groups were observed in Accuracy and the Impulsivity Control Index (ICI). The STEAM group scored higher than the control group on Accuracy (*p* = 0.000) and on the ICI (*p* = 0.000).

## 4. Discussion

The present study aimed to explore whether executive functions and cognitive skills in children with ADHD were related to participation in an educational program based on the STEAM methodology. The program included activities such as robotics, computational thinking, coding, and programming, in line with previous research indicating that practical, collaborative, and hands-on experiences can strengthen working memory, inhibition, planning, and other executive functions, as well as general cognitive abilities [28,30,31,32,34,38].

Children in the STEAM group tended to achieve better performance across several cognitive and executive domains compared to the control group. These differences have significant implications for the daily lives of children with ADHD, as more developed executive functions are associated with better academic outcomes, greater autonomy in managing everyday tasks, more effective emotional regulation, and more adaptive social interactions [7,8,15,16].

On the BRIEF, the STEAM group showed lower scores, which on the BRIEF scale indicates better executive functioning in almost all subscales. Specifically, in executive functions such as inhibition, planning, and organization, statistical significance was achieved. These findings are consistent with previous studies that reported improvements in specific executive functions, such as inhibition [31] and planning [35]. Moreover, other authors working with targeted samples of children diagnosed with ADHD have documented significant gains in inhibition and planning following STEAM-based interventions [41]. For children with ADHD, improved inhibition can reduce impulsive responses and enhance decision-making in social and academic contexts, while gains in planning facilitate the organization of school tasks, adherence to routines, and execution of multi-step activities [18,19,20]. Strengthening these domains can also foster emotional competence, supporting more positive peer relationships and greater resilience [15,16]. However, in our study, children in the control group obtained better scores on the flexibility subscale, and these differences reached statistical significance, with an effect size approaching the moderate range. This finding contrasts with previous research, conducted with typically developing students [38], where significant differences were observed in favor of the group participating in STEAM-based interventions.

The remaining BRIEF scales did not yield statistically significant differences, as was the case with working memory. This contrasts with findings from other authors, such as [33], who reported significant improvements in their studies with larger samples composed of typically developing children. The fact that some score differences did not reach statistical significance, or that significance favored the control group—contrary to what was expected—may be due to the limited sample size used in the present study, as well as the exclusive inclusion of children diagnosed with ADHD.

Regarding WISC-V measures, the results suggest that, on average, children who participated in the STEAM program performed better across all cognitive domains assessed by the WISC-V. The STEAM group demonstrated significantly higher performance in verbal comprehension, visuospatial skills, and processing speed, as well as a higher total IQ. Furthermore, the effect sizes observed through Cohen’s d indicate that the STEAM intervention indicates large effect sizes associated with participation that influence verbal, visuospatial, processing speed and overall intellectual functioning in children with ADHD. Specifically, a Cohen’s d of 1.28 was obtained for visuospatial performance, indicating a very strong effect. This finding aligns with previous studies [30,32] that have reported significant improvements in visuospatial abilities following the implementation of STEM-based methodologies. These results suggest that STEAM may be associated with both specific executive functions and broader cognitive abilities, in line with previous research on the cognitive benefits of robotics, programming, and computational thinking [28,30,31,33,34,38].

From a functional perspective, stronger verbal comprehension facilitates following complex instructions, understanding rules, participating actively in class, and adapting to new contexts [11]. Well-developed visuospatial skills support tasks requiring spatial reasoning, organization of materials, and navigation of environments, contributing to greater independence [13]. Faster processing speed enables children to complete academic and everyday tasks more efficiently, reducing frustration and cognitive overload [12]. These cognitive improvements are linked to better planning, attention, and use of adaptive strategies [7,10], as well as enhanced emotional regulation and social competence [8,15,16].

Similarly, the CARAS-R results revealed significant differences in accuracy and impulse control, with the STEAM group outperforming the control group. For the Accuracy measure, the results indicate better performance in the STEAM group. Also, regarding the Impulsivity Control Index (ICI), the results suggest greater impulsivity control in the STEAM group. Significant differences between groups were observed in Accuracy and the Impulsivity Control Index (ICI), indicating better performance in both measures. Higher scores on both Accuracy and the ICI reflect better attentional control and lower impulsivity in the STEAM group compared to controls. These results are consistent with recent findings [41], who reported significant improvements in sustained attention, inhibition, and impulse control in a targeted sample of children diagnosed with ADHD following the implementation of STEAM-based interventions. This reinforces the idea that STEAM-based interventions can promote sustained attention and inhibition, competencies often compromised in children with ADHD [4,5,6]. As some authors [14] note, greater self-control in early childhood predicts more effective peer relationships, adherence to rules and routines, and better decision-making during the transition to adulthood, which could be related to the differences in executive functioning observed in our study.

The results obtained across the different instruments converge, reinforcing the consistency of the findings. In the BRIEF, statistically significant differences favored the STEAM group in inhibition, which aligns with the significant differences observed in the CARAS-R for Accuracy and the Impulsivity Control Index (ICI), both directly related to impulse control. Similarly, the STEAM group showed better performance in the WISC-V Processing Speed Index, a domain associated with attentional efficiency and inhibitory control, further supporting the CARAS-R outcomes. Finally, the STEAM group achieved higher scores in planning and organization on the BRIEF, which corresponds with their superior performance in the WISC-V Visuospatial Index, a measure related to organizational and spatial reasoning skills [46,48,51].

Children with ADHD often report multiple difficulties, such as inattention and impulsivity [1,2]. Therefore, the significant difference observed in indicators related to inhibition and attention, such as the CARAS-R and the inhibition scale of the BRIEF, is particularly relevant. In addition, children with ADHD frequently present language difficulties [5], which underscores the importance of the better performance shown by the experimental group in verbal tasks. Altogether, these advances reflect a more efficient executive functioning profile that translates into enhanced learning capacities [18,19,20,21].

Taken together, these findings suggest that participation in STEAM may be associated with both general cognition and specific executive functions, including flexibility, inhibition, planning, and attention. The observed trends support the hypothesis that STEAM can be a valuable tool for fostering cognitive and executive skills in children with ADHD. This is consistent with the literature, which highlights that active, hands-on, and collaborative methodologies can promote the development of cognitive competencies [22,24,25].

Therefore, while the results suggest promising associations between STEAM participation and differences in executive and cognitive skills, they should be interpreted cautiously. Future research should employ randomized controlled trials or longitudinal designs to better establish causal relationships.

Despite these promising results, the relatively small sample size remains a limitation, which may have reduced statistical power. Larger studies are needed to confirm these findings and more precisely estimate the associations related to STEAM interventions and cognitive development in children with ADHD.

## 5. Conclusions

The results of the present study suggest associations between participation and higher scores in executive functions and cognitive skills, which in turn may lead to improvements in their academic performance, school adaptation, and quality of life. However, given the absence of pretest data to ensure initial equivalence between groups, these conclusions should be interpreted with caution. Enhancements in areas such as inhibition, working memory, processing speed, and visuospatial abilities translate into greater autonomy in meeting daily demands, more adaptive social interactions, and more effective emotional regulation. The sustained and systematic integration of STEAM activities has been highlighted in the previous literature as an ideal context for stimulating these abilities through motivating, practical, and collaborative experiences. Prior research also suggests that, beyond cognitive and executive development, this approach also has clear pedagogical implications, as it promotes inclusive practices that foster impulse control, flexible thinking, planning, and language skills, thereby supporting classroom participation and academic achievement in children with ADHD. Future studies with larger and more diverse samples, as well as longitudinal designs, are needed to confirm these findings, explore additional executive and cognitive domains, and assess the long-term educational benefits of STEAM interventions. A major limitation of this study is the use of a non-equivalent control group, which introduces potential self-selection bias. Families who choose STEAM programs may differ in relevant ways from those who do not, and the longer average exposure time of the STEAM group further constrains causal interpretation. In addition, it is recommended that future research include pretest measures or other procedures to ensure group equivalence prior to intervention, taking into account social, economic, and contextual factors that may affect the data and potentially alter the interpretation of results. Finally, the considerable variability in the duration of exposure to the STEAM program (M = 3.93 years, SD = 1.28) was not statistically controlled for, which may have influenced the outcomes. Future studies should account for exposure length to better examine potential dose–response effects. Moreover, future research should also investigate the interplay between executive functions and social skills in students with ADHD, as this could provide a more comprehensive understanding of the educational and socio-emotional impact of STEAM interventions.

## Figures and Tables

**Table 1:** Descriptive statistics of participants by group.

Group	N	Age (MD)	Boys	Girls	Years in STEAM (MSD)
Control	30	8.47 (2.232)	22	8	-
STEAM	30	8.53 (2.356)	22	8	1.280

**Table 2:** Results between STEAM and control groups (BRIEF).

BRIEF Subscale	Group	N	Mean	SD	Z	*p*	d de Cohen
Inhibition	Control	30	61.13	12.216	−2194	0.028 *	0.61
	STEAM	30	54.60	8.920			
Self-monitoring	Control	30	56.40	10.074	−1906	0.057	0.42
	STEAM	30	60.80	10.714			
Flexibility	Control	30	56.93	12.151	−1961	0.050 *	0.46
	STEAM	30	62.80	13.319			
Emotional control	Control	30	54.40	13.559	−0.059	0.953	0.03
	STEAM	30	53.93	11.688			
Initiative	Control	30	56.07	12.309	−0.148	0.882	0.12
	STEAM	30	57.53	12.278			
Working memory	Control	30	62.53	12.784	−0.948	0.343	0.19
	STEAM	30	59.80	14.981			
Planning and organization	Control	30	61.27	12.208	−1983	0.047 *	0.42
	STEAM	30	56.00	12.523			
Task supervision	Control	30	59.47	12.849	−1008	0.314	0.16
	STEAM	30	57.33	13.780			
Organization of materials	Control	30	55.93	13.075	−0.267	0.789	0.03
	STEAM	30	56.33	12.604			

Mann–Whitney U test results for BRIEF between STEAM and control groups. * Statistical significance was defined as *p* < 0.05.

**Table 3:** Results between STEAM and control groups (WISC-V).

WISC-V Index	Group	N	Mean	SD	t	*p*	d de Cohen
Visuospatial Index	Control	30	97.07	9.017	−4.975	0.000 *	1.28
	STEAM	30	114.33	16.736			
Fluent Reasoning Index	Control	30	101.80	9.953	−1.636	0.107	0.42
	STEAM	30	107.40	15.891			

Independent samples *t*-test results for WISC-V between the STEAM and control groups. * Statistical significance was defined as *p* < 0.05.

**Table 4:** Results between STEAM and control groups (WISC-V).

WISC-V Index	Group	N	Mean	SD	Z	*p*	d de Cohen
Verbal Comprehension Index	Control	30	101.47	14.493	−2.524	0.012 *	0.79
	STEAM	30	112.13	12.097			
Working Memory Index	Control	30	98.13	14.137	−0.237	0.812	0.17
	STEAM	30	101.00	19.093			
Processing Speed index	Control	30	81.80	12.257	−2.757	0.006 *	0.79
	STEAM	30	92.67	14.700			
Total IQ	Control	30	95.27	7.625	−3.620	0.000 *	1.05
	STEAM	30	106.73	13.128			

Mann–Whitney U test results for WISC-V between the STEAM and control groups. * Statistical significance was defined as *p* < 0.05.

**Table 5:** Results between STEAM and control groups (CARAS-R).

CARAS-R Measures	Group	N	Mean	SD	Z	*p*	d de Cohen
Accuracy	Control	30	3.13	1.570	−4.046	0.000 *	1.26
	STEAM	30	5.20	1.710			
Impulsivity Control Index	Control	30	2.40	1.522	−4.679	0.000 *	1.46
	STEAM	30	4.53	1.383			

Mann–Whitney U test results for CARAS-R showing significant differences between STEAM and control groups. * Statistical significance was defined as *p* < 0.05.

## Data Availability

The data presented in this study are available on request from the corresponding author. The datasets presented in this article are not immediately available, as they are part of an ongoing research study. Requests for access to the datasets should be addressed to the corresponding author of the article with prior permission from the R&D center where the study was conducted.

## Abbreviations

The following abbreviations are used in this manuscript:

ADHD	Attention Deficit Hyperactivity Disorder
STEAM	Science, Technology, Engineering, Art and Mathematics.
EFs	Executive Functions
BRIEF	Behavior Rating Inventory of Executive Functions
WISC-V	Wechsler Intelligence Scale for Children, 5º edition
IQ	Intelligence Quotient
ICI	Impulsivity Control Index
